# Association between *LRRK2* gene polymorphisms and Parkinson’s disease progression in a Chinese Han population

**DOI:** 10.3389/fgene.2025.1662348

**Published:** 2025-09-25

**Authors:** Zhaoting Zhang, Lei Geng, Jiuxin Gao, Ruifang She, Zhonglin Ge, Jianhua Liu, Qianqian He, Bing Fu, Weiguo Liu

**Affiliations:** ^1^ Department of Neurology, The Second People’s Hospital of Lianyungang, Lianyungang, Jiangsu, China; ^2^ Department of Radiology, The Second People’s Hospital of Lianyungang, Lianyungang, Jiangsu, China; ^3^ Department of Neurology, Tai’an Central Hospital Affiliated to Qingdao University, Tai’an, Shandong, China; ^4^ Department of Neurology, Nanjing Brain Hospital Affiliated to Nanjing Medical University, Nanjing, Jiangsu, China

**Keywords:** Parkinson’s disease risk, LRRK2 polymorphisms, disease progression, disease severity, genetic association study

## Abstract

**Objective:**

The objective of this study was to investigate the association between *LRRK2* gene polymorphisms and Parkinson’s disease (PD) risk, as well as the progression of PD, in a Chinese Han population.

**Methods:**

A case-control study was carried out on 180 PD patients and 196 healthy controls between October 2019 and October 2023. The demographic and clinical data of all participants were collected. At the baseline and 3-year follow-up, assessment of motor and non-motor symptoms of PD patients were carried out using scales including Unified Parkinson’s Disease Rating Scale parts II, III, and IV, Hoehn and Yahr (H-Y) staging scale, Hamilton Depression Rating Scale, Hamilton Anxiety Rating Scale, Non-motor Symptom Questionnaire, Parkinson’s disease sleep scale, Montreal Cognitive Assessment, and Mini-Mental State Examination. Six single nucleotide polymorphisms (SNPs) of the *LRRK2* gene rs1994090, rs2046932, rs2708453, rs34778348, rs4768212, and rs7304279 were selected and genotyped using the MassARRAY platform in all participants.

**Results:**

A strong linkage disequilibrium was observed among the five SNP loci of the *LRRK2* gene, including rs1994090, rs2046932, rs2708453, rs4768212, and rs7304279. *LRRK2* rs7304279 (OR = 3.572, P < 0.001) and rs34778348 (OR = 0.408, P = 0.003) contributed to the risk of PD development. Carriage of more risk variants were associated with higher risk of PD (OR = 6.467, P < 0.001). Cox proportional hazards model analysis showed that *LRRK2* rs7304279 polymorphism was significantly associated with H-Y stage progression (*P* = 0.030).

**Conclusion:**

Our findings suggest that *LRRK2* rs34778348 and rs7304279 polymorphisms are associated with the risk of developing PD. And *LRRK2* rs7304279 polymorphism is correlated with disease progression in PD patients.

## 1 Introduction

Parkinson’s disease (PD) is the second most common neurodegenerative disease in the middle-aged and elderly population, affecting 1-2 per 1,000 of the population (Sardi et al., 2018). Most PD patients are sporadic, likely arising from a combination of polygenic inheritance, environmental exposures and gene-environment interactions. Leucine-rich repeat kinase 2 (*LRRK2*) gene mutations are the major genetic cause of late-onset autosomal-dominant PD (Nabli et al., 2015). The LRRK2 protein encoded by the *LRRK2* gene is an intracellular signaling protein involved in many physiological functions, including substrate binding, protein phosphorylation and protein-protein interaction (Mata et al., 2006). The LRRK2 protein can also function as a GTPase and protein kinase activities that may contribute to pathogenicity (Taymans and Cookson, 2010), and lead to the development of PD. It has been shown that *LRRK2* is involved in a wide range of cellular functions including neurite growth, maintenance of cytoskeleton, vesicular transport, autophagy, protein degradation, inflammation, and immune response (Calogero et al., 2019). Genome-wide association study (Chang et al., 2011) showed that alleles at the loci such as rs1994090, rs2046932, and rs7304279 in the *LRRK2* gene were significantly higher in PD patients compared to healthy controls. The single nucleotide polymorphism (SNP) in *LRRK2* G2385R (rs34778348) can encode a large complex protein with two enzymatic activities (Russo, 2019), which increases the prevalence of PD in Chinese populations by 2 times (Wu-Chou et al., 2013).

Genes can interact with each other and/or interact with environmental factors, and linkage disequilibrium may exist among different SNP loci (Li et al., 2019a). Previous studies (Chang et al., 2011; Kim et al., 2010) have primarily focused on the impact of single or dual genetic loci within LRRK2 gene on the risk of developing PD, but the interactions among multiple loci within LRRK2 in Chinese patients with PD are rarely studied. Therefore, this study aimed to investigate whether the six SNPs (rs1994090, rs2046932, rs2708453, rs34778348, rs4768212, and rs7304279) in the *LRRK2* gene were associated with sporadic PD in a population from China, and explore whether PD-risk SNPs were associated with the severity and progression of PD in Chinese Han population.

## 2 Materials and methods

### 2.1 Participants

A total of 180 PD patients who attended Nanjing Brain Hospital and The Second People’s Hospital of Lianyungang between October 2020 and October 2023 were included. The inclusion criteria included: patients who met the International Parkinson and Movement Disorder Society clinical diagnostic criteria for PD published in 2015 (Postuma et al., 2015); the diagnosis of PD was made independently by two experienced neurologists specializing in PD. The exclusion criteria included: patients who had no personal or family history of intracranial organic diseases, psychiatric diseases, severe systemic diseases, severe cognitive dysfunction, extrapyramidal diseases (such as secondary parkinsonism).

The control group consisted of 196 healthy individuals who underwent physical examination in Nanjing Brain Hospital and The Second People’s Hospital of Lianyungang during the study period. Healthy controls were matched to PD patients for age, gender, and regional distribution.

All participants or their guardians provided written informed consent. The study was approved by the Ethics Committee of Nanjing Medical University.

### 2.2 Methods

#### 2.2.1 Clinical data collection

At baseline and 3-year follow-up, PD patients who met the inclusion and exclusion criteria were assessed by two well-trained neurologists. All PD patients completed the relevant assessment scales during the “off” state. Clinical data including patients’ demographic data, previous medical history, detailed neurological physical examination results, and results from PD-related scales were collected and recorded.

#### 2.2.2 Assessment of motor symptoms

At baseline and 3-year follow-up, PD patients were assessed using the Unified Parkinson’s Disease Rating Scale (UPDRS) parts II, III, and IV. The Hoehn and Yahr (H-Y) staging scale was used to assess the severity of PD (Chen et al., 2016).

#### 2.2.3 Assessment of non-motor symptoms

At baseline and 3-year follow-up, the non-motor symptoms of patients were evaluated by the following scales: Montreal Cognitive Assessment (MOCA), Mini-Mental State Examination (MMSE), Non-motor Symptom Questionnaire (NMSQ), Hamilton Depression Rating Scale (HAMD), Hamilton Anxiety Rating Scale (HAMA), and the Parkinson’s disease sleep scale (PDSS).

#### 2.2.4 Follow-up and endpoints

All patients were followed up for 3 years. Endpoints included loss to follow-up and death of PD patients.

#### 2.2.5 SNPs selection and genotyping

Peripheral venous blood (2–3 mL) from all participants was collected into EDTA anticoagulated tubes, and stored at −70 °C. The genomic DNA was extracted from peripheral blood samples using chloroform method. Six SNPs of the *LRRK2* gene including rs1994090, rs2046932, rs2708453, rs34778348, rs4768212, and rs7304279 were selected and sequenced using Paired-end sequencing. Primers were designed by Genotyping Tools and MassARRAY Assay Design software (Sequenom, San Diego, CA, United States). Multiplexed PCR amplification was then performed. The PCR reaction conditions were as follows: 94 °C for 4 min; 94 °C for 20 s, 56 °C for 30 s, and 72 °C for 1 min, 45 cycles; 72 °C for 3 min; and 4 °C forever. Base pair sequencing was performed using the microarray method to determine the base pairs at the loci. Genotypes were analyzed using Typer 4.0 software (Sequenom, San Diego, CA, United States).

### 2.3 Statistical analysis

Statistical analyses were performed using SPSS statistical software version 27.0. Continuous variables were expressed as mean ± standard deviation (SD). Normally distributed continuous data was analyzed by t-test, and non-normally distributed continuous data analyzed by non-parametric Mann-Whitney U test. The differences were considered significant at P < 0.05. Categorical variables were expressed as rate or constituent ratio, and analyzed using the χ^2^ test. The χ^2^ test was used to test whether the distribution of the observed genotypes in the different groups followed the Hardy-Weinberg equilibrium. P > 0.05 suggests that the sample provides a good representation of the population and genotype frequencies was in agreement with Hardy-Weinberg equilibrium. χ^2^ test was used to compare allele and genotype frequencies between PD patients and healthy controls, and P < 0.05 was considered statistically significant. The P-value was corrected for multiple testing using Bonferroni correction. The odds ratio (OR) and 95% confidence interval (CI) were used to assess the relative risk. Linkage disequilibrium between all SNP pairs, measured as *r* ^2^, was estimated by PLINK v.1.9 software, and visualized by LDBlockshow software (Dong et al., 2021). r^2^ ≥ 0.8 indicates that the SNPs are in strong LD. χ^2^ test was used to calculate the cumulative effects of multiple risk-associated SNPs on the risk of PD. Cox proportional hazards model was used to analyze the correlation between the PD risk-associated SNPs and the progression of PD, and a P ≤ 0.05 was considered statistically significant.

## 3 Results

### 3.1 Demographic characteristics of PD patients and healthy controls

This study included a total of 180 PD patients and 196 healthy controls. In the patient group, there were 103 (57.22%) males and 77 (42.78%) females, with an average age of 68.82 ± 10.57 years. In the healthy control group, there were 107 (54.59%) males and 89 (45.41%) females, with an average age of 67.26 ± 5.57 years. The two groups were matched for age (P = 0.071) and gender (P = 0.678), and the differences between the two groups were not statistically significant.

### 3.2 Clinical characteristics of PD patients at baseline and follow-up

Among 180 PD patients included at baseline, 20 patients were lost to follow-up and died, 160 patients were finally followed up for 3 years. Compared with the baseline, PD patients showed significantly higher scores of rigidity, bradykinesia, and axial symptoms, significantly higher UPDRS II, UPDRS Ⅲ, UPDRS IV, HAMD, and NMSQ scores, significantly lower PDSS, MMSE and MoCA scores, as well as significantly higher H-Y stage at the 3-year follow-up (all P < 0.05, Table 1).

**TABLE 1:** Comparison of patient demographics and clinical characteristics at baseline and follow-up.

Clinical characteristics	Baseline (n = 180)	Follow-up (n = 160)	*t*/*z*	*P* value
Tremor score	4.97 ± 3.17	5.09 ± 4.13	−0.374	0.709
Rigidity score	3.70 ± 3.38	6.37 ± 4.16	−7.114	**<0.001**
Bradykinesia score	8.74 ± 5.71	11.57 ± 6.64	−5.186	**<0.001**
Axial symptom score	4.16 ± 2.73	5.81 ± 3.91	−5.215	**<0.001**
HAMD score	12.43 ± 9.81	14.79 ± 10.04	−3.008	**0.003**
HAMA score	10.13 ± 7.74	11.42 ± 7.58	−1.862	0.065
PDSS score	119.36 ± 20.51	113.85 ± 23.92	3.104	**0.002**
NMSQ score	10.30 ± 5.27	12.61 ± 5.64	−5.924	**<0.001**
MMSE score	28.29 ± 2.01	26.63 ± 3.97	6.178	**<0.001**
MOCA score	24.16 ± 4.37	22.26 ± 5.83	5.409	**<0.001**
UPDRS II score	11.96 ± 5.84	14.67 ± 7.38	−5.197	**<0.001**
UPDRS III score	22.46 ± 12.05	31.79 ± 15.86	−7.383	**<0.001**
UPDRS IV score	2.30 ± 1.42	3.55 ± 2.54	−4.842	**<0.001**
H-Y stage	2.0 (1.5,2.5)	2.0 (1.5,2.5)	−9.820	**<0.001**

HAMD, hamilton depression rating scale; HAMA, hamilton anxiety rating scale; PDSS, the Parkinson’s disease sleep scale; NMSQ, Non-Motor Symptoms Questionnaire; MMSE, Mini-Mental State Examination; MOCA, montreal cognitive assessment; UPDRS II, the Unified Parkinson’s Disease Rating Scale part II; UPDRS III, the Unified Parkinson’s Disease Rating Scale part III; UPDRS IV, the Unified Parkinson’s Disease Rating Scale part IV; H-Y stage, Hoehn and Yahr stage.

Data were analyzed using rank sum test. p < 0.05 indicates statistically significant. The bold values indicate statistically significant results.

### 3.3 Hardy-weinberg equilibrium test results

Hardy-Weinberg equilibrium test was performed for the genotypes of the six *LRRK2* SNPs (rs1994090, rs2046932, rs2708453, rs34778348, rs4768212, and rs7304279) in PD patients and healthy controls. The genotype frequencies can be calculated from actual observed allele frequencies. The expected genotype frequencies can be obtained by multiplying the frequencies with the total population number. χ^2^ test was used to analyze the differences between the observed and expected genotype frequencies. The results showed that in both the patient and healthy control groups, genotypic distributions of the six SNPs followed the Hardy-Weinberg equilibrium with a p-value >0.05. The results indicate that the sample is in Hardy-Weinberg genetic equilibrium, which is a good representation of the population.

### 3.4 Linkage disequilibrium genetic analysis results

**FIGURE 1:**
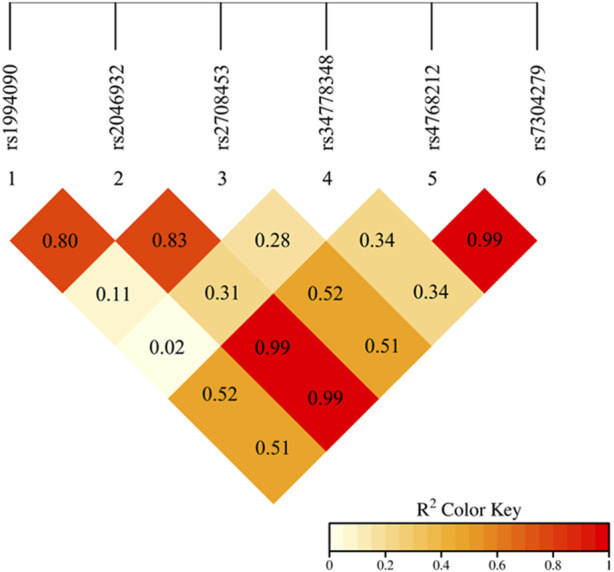
Linkage disequilibrium analysis of the alleles of six SNPs in the *LRRK2* gene. The red color in the box indicates that five SNPs have strong LD (*r* ^2^ ≥ 0.8).

Linkage disequilibrium (estimated as the *r* ^2^) between alleles of the six *LRRK2* SNPs were calculated by PLINK software. An *r* ^2^ value of ≥0.8 represents the SNPs were in strong LD. The results showed that there was strong linkage disequilibrium among the five SNPs in the *LRRK2* gene, including rs1994090, rs2046932, rs2708453, rs4768212, and rs7304279 (Figure 1).

### 3.5 Associations of *LRRK2* SNPs with PD risk

Based on the linkage disequilibrium genetic analysis results, 4 SNPs (rs1994090, rs2046932, rs2708453, and rs4768212) with strong linkage disequilibrium were removed, then the correlation between SNPs rs34778348, rs7304279 and the risk of PD was assessed by χ^2^ test. The results showed that the genotype and allele frequencies of rs34778348 and rs34778348 differed significantly between PD patients and healthy controls (all P < 0.05, Table 2).

**TABLE 2:** Genotype and allele frequencies of *LRRK2* rs34778348 and rs7304279 in PD patients and healthy controls (n/%).

SNP	Genotype allele	PD patients (n = 180)	Controls (n = 196)	χ2 value	OR (95%CI)	*P* value
rs34778348	A	34 (9.44%)	16 (4.08%)	**8.695**	0.408 (0.221–0.753)	0.003
	G	326 (90.56%)	376 (95.92%)			
	AA	1 (0.55%)	0 (0%)	**8.999**		0.011
	AG	32 (17.78%)	16 (8.16%)			
	GG	147 (81.67%)	180 (91.84%)			
rs7304279	T	58 (16.11%)	20 (5.50%)	**24.465**	3.572 (2.101–6.071)	<0.001
	C	302 (83.89%)	372 (94.90%)			
	TT	8 (4.45%)	1 (0.51%)	**21.598**		<0.001
	CT	42 (23.33%)	18 (9.18%)			
	CC	130 (72.22%)	177 (90.31%)			

PD, Parkinson’s disease; SNPs, single nucleotide polymorphisms.

P *<* 0.05 indicates significant association. The bold values indicate statistically significant results.

### 3.6 Cumulative risk analysis of PD risk-associated SNPs

*LRRK2* rs34778348 A allele and rs7304279 T allele were associated with increased PD risk (P < 0.05), these variants were pathogenic. Among the 180 PD patients, 123 carried 0 risk variant, 31 carried 1 risk variant, and 26 carried 2 risk variants. Among 196 healthy controls, 177 carried 0 risk variant, 12 carried 1 risk variant, and 7 carried 2 risk variants. The chi-square test was used to compare the differences in PD risk between PD patients and healthy controls carrying 0 variant and those carrying 1 and 2 risk variants. The results showed that the more the number of risk allele carried, the higher the risk of PD (OR = 6.467, P < 0.001, Table 3).

**TABLE 3:** Cumulative effects of PD risk-associated SNPs on PD risk among patients and healthy controls with 0, 1 and 2 risk variants.

The number of risk variant carried	PD patients (n = 180)	Controls (n = 196)	*P* value	χ2	OR	95%CI
0	123 (68.33%)	177 (90.31%)				
1	31 (17.22%)	12 (6.12%)	<0.001	18.499	4.872	(2.240–10.600)
2	26 (14.45%)	7 (3.57%)	<0.001	22.908	6.467	(2.764–15.127)

PD, Parkinson’s disease; SNPs, single nucleotide polymorphisms.

p < 0.05 indicates statistically significant.

### 3.7 Risk factors for H -Y stage progression

According to changes in H-Y stage at 3-month follow-up, final follow-up patients were divided into a stable group (changes from baseline to follow up in H-Y stage ≤0, assigned a value of 0) and a progressive group (changes from baseline to follow up in H-Y stage >0, assigned a value of 1). The variables including gender, age of onset, baseline tremor, rigidity, bradykinesia score, axial symptom scores, baseline UPDRS II, UPDRS Ⅲ, HAMD, HAMA, PDSS, NMSQ, MOCA, MMSE scores, baseline H-Y stage, *LRRK2* rs34778348, and rs7304279 polymorphisms were subjected to univariate analysis (Table 4). Variables with statistical significance (p < 0.05) in the univariate analysis were included in the Cox proportional hazards model. The results revealed that *LRRK2* rs7304279 polymorphism was significantly associated with H-Y stage progression (*P* = 0.030, Table 5).

**TABLE 4:** Univariate analysis of factors associated with H &Y stage progression.

Variables		Stable group (n = 71)	Progressive group (n = 89)	*t*/χ2	P value
Geder (male/female)		39/32	55/34	0.769	0.381
Age at disease onset		57.72 ± 10.00	58.55 ± 12.62	−0.446	0.656
Baseline tremor score		5.05 ± 3.65	5.12 ± 3.20	−0.120	0.904
Baseline rigidity score		4.96 ± 3.73	2.81 ± 2.78	3.976	**<0.001**
Baseline bradykinesia score		9.22 ± 5.88	8.25 ± 5.47	1.027	0.306
Baseline axial symptom score		4.44 ± 2.99	4.06 ± 2.60	0.807	0.421
Baseline HAMD score		12.47 ± 9.01	12.40 ± 10.47	0.047	0.962
Baseline HAMA score		10.26 ± 6.94	10.03 ± 8.38	0.174	0.862
Baseline PDSS score		116.18 ± 23.41	121.74 ± 17.79	−1.688	0.093
Baseline NMSQ score		9.63 ± 4.79	10.84 ± 5.59	−1.441	0.151
Baseline MMSE score		27.86 ± 2.28	28.60 ± 1.74	−2.269	**0.025**
Baseline MOCA score		23.32 ± 4.87	24.85 ± 3.79	−2.162	**0.032**
Baseline UPDRS II score		11.84 ± 5.23	12.06 ± 6.39	−0.206	0.837
Baseline UPDRS III score		24.90 ± 11.91	20.61 ± 11.90	2.154	**0.033**
Baseline UPDRS IV score		2.30 ± 1.27	2.31 ± 1.51	−0.053	0.958
rs34778348	AA	1 (1.41%)	0 (0%)	3.686	0.158
	AG	9 (12.68%)	20 (22.47%)		
	GG	61 (85.91%)	69 (77.53%)		
rs7304279	TT	6 (8.45%)	2 (2.25%)	13.901	**<0.001**
	CT	9 (12.68%)	33 (37.08%)		
	CC	56 (78.87%)	54 (60.67%)		

HAMD, hamilton depression rating scale; HAMA, hamilton anxiety rating scale; PDSS, the Parkinson’s disease sleep scale; NMSQ, Non-Motor Symptoms Questionnaire; MMSE, Mini-Mental State Examination; MOCA, montreal cognitive assessment; UPDRS II, the Unified Parkinson’s Disease Rating Scale part II; UPDRS III, the Unified Parkinson’s Disease Rating Scale part III; UPDRS IV, the Unified Parkinson’s Disease Rating Scale part IV; H-Y stage, Hoehn and Yahr stage. The bold values indicate statistically significant results.

**TABLE 5:** Cox proportional hazards model analysis of risk factor for H &Y stage progression.

Risk factor	B	Standard error	P	HR value	95%CI
Rigidity score	−0.014	0.031	0.654	0.986	(0.928–1.048)
UPDRS III score	−0.004	0.009	0.650	0.996	(0.979–1.013)
MMSE score	0.056	0.084	0.503	1.058	(0.897–1.248)
MOCA score	0.001	0.037	0.983	1.001	(0.930–1.076)
rs7304279			0.030		
rs7304279 (CC)	−0.052	0.745	0.945	0.950	(0.221–4.090)
rs7304279 (CT)	0.589	0.743	0.428	1.803	(0.420–7.737)

MMSE, Mini-Mental State Examination; MOCA, montreal cognitive assessment; UPDRS III, the Unified Parkinson’s Disease Rating Scale part III; H-Y stage, Hoehn and Yahr stage.

p < 0.05 indicates statistically significant.

## 4 Discussion

This study demonstrated a significant association of *LRRK2* rs34778348 and rs7304279 with risk for PD in a Chinese Han population, as well as a correlation between rs7304279 polymorphism and PD progression. These findings provide valuable insights into the genetic architecture of PD, shed light onto the pathogenesis of PD, and may facilitate the development of precise strategies for the prevention, diagnosis, and treatment of PD.

PD is a complex neurodegenerative disease with diverse clinical manifestations, including motor symptoms such as resting tremor and rigidity, and non-motor symptoms such as hyposmia, sleep disorders, autonomic dysfunction, mood disorders, and cognitive deficits (Poewe, 2008). There are individual differences in the clinical symptoms and disease progression among PD patients (Schrag et al., 2006). In this study, a total of 180 patients were enrolled at baseline and 160 patients were finally followed up. Comparison of the clinical characteristics of PD patients at baseline and 3-year follow-up showed that with disease progression, significant differences in the scores of rigidity, bradykinesia, axial symptom, HAMD, PDSS, NMSQ, MMSE, MoCA, UPDRS II, UPDRS Ⅲ, and UPDRS IV scores, as well as H-Y stage were observed between baseline and follow-up in PD patients (all P < 0.05). However, the tremor score did not differ significantly between baseline and 3-year follow-up (P = 0.709). The results suggest that the progression of tremor symptoms in PD patients is relatively slow, rigidity, bradykinesia, and axial symptoms have progressed. This may be due to different pathophysiologic processes for different symptoms. Resting tremor is the result of the interaction between the basal ganglia and the cerebello-thalamo-cortical circuit (Mure et al., 2011), while rigidity and bradykinesia are only associated with the basal ganglia (Xu et al., 2018). Axial symptoms are associated with the pontine nuclei (Xiao et al., 2017). Excessive synchronization between basal ganglia neurons contributes to the clinical features of PD. Tremor symptoms in PD patients are associated with neuronal activity in the centromedial_parafascicular nuclei of the thalamus (McGregor and Nelson, 2019), as well as synchronous neuronal oscillations in the globus pallidus externus and globus pallidus internus (Levy et al., 2002). With the progression of the disease, the synchronization mechanism may be destroyed, and the worsening of tremor symptoms is not apparent.

This study revealed that five SNP loci of the *LRRK2* gene, including rs1994090, rs2046932, rs2708453, rs4768212, and rs7304279, were in strong linkage disequilibrium (r2 ≥ 0.8). The result is consistent with findings from Satake et al. (2009). The results of this study showed that there were significant differences in the genotype and allele frequencies of the two *LRRK2* SNPs rs34778348 (OR = 0.408, P = 0.003) and rs7304279 (OR = 3.572, P < 0.001) between PD patients and healthy controls. The results were also consistent with studies of Park et al. (2023) and Liu et al. (2020) showing that the two SNPs rs34778348 and rs7304279 were associated with the risk of developing PD. *LRRK2* variant rs34778348 can block the dimerization of LRRK2 WD40 domain (Kalogeropulou et al., 2022), and disrupt intracellular vesicle transport (Carrion et al., 2017). *LRRK2* kinase activity-mediated cohesion deficits are commonly seen in carriers of different LRRK2 mutations (Naaldijk et al., 2024). These may represent the pathophysiological basis for the development of PD.

Our finding showed that carriage of more risk variants were associated with increased risk for PD (OR = 6.467, P < 0.001). Yu et al. (2015) reported that the risk of PD gradually increased with the increasing number of risk variants carried by individuals, and the risk variants have a cumulative risk effect on increased PD susceptibility. Li et al. (2019b) also showed that PD is a disease that can be attributable to multiple genes rather than a single gene.

In this study, final follow-up patients were divided into stable and progressive groups according to the change in H-Y stage at follow-up. Univariate analysis identified baseline rigidity, UPDRS III, MMSE, MOCA scores, and rs7304279 as significant risk factors for H-Y stage progression (P < 0.05). These variables were then subjected to Cox proportional hazards model. The results showed that rs7304279 polymorphism in the *LRRK2* gene was correlated with H-Y stage progression (P = 0.030). This is consistent with the finding from a study conducted by Yu et al. (2015). The rs7304279 locus is located from intron 2 of SLC2A13 to 38.4 kb upstream of *LRRK2*, which may be associated with transcriptional upregulation of the *LRRK2* gene (Mure et al., 2011), increases kinase activity of mutant *LRRK2* to mediate neuronal toxicity (Smith et al., 2006), thus leading to a loss of dopaminergic neurons. In this study, univariate analysis showed that the rigidity score at baseline was significantly higher in the stable group than that in the progressive group (P < 0.001), and the lower the baseline rigidity score, the faster the progression of PD. However, the association between rigidity score and H-Y stage progression was not observed using Cox proportional hazards model analysis.

Limitations of this study include small sample size and short duration of follow up. Furthermore, due to the small sample size, it was not feasible to further explore the association between SNPs and non-motor symptoms in PD. Further studies with larger sample size and longer follow-up periods are needed to not only confirm the findings, but also to conduct a more comprehensive investigation into the association between SNPs and non-motor symptoms.

## 5 Conclusion

In conclusion, our findings suggest that two SNPs rs34778348 and rs7304279 in the *LRRK2* gene are associated with the risk of developing PD, and *LRRK2* SNP rs7304279 is correlated with the disease progression of PD. The development of PD is caused by gene-environment interactions. This emphasizes the importance of genetic factors in the development of PD, which is pivotal for the development of targeted therapies.

## Data Availability

The data cannot be shared openly to protect study participant privacy. However, the data can be made available from the authors upon reasonable request.
